# Evolving understanding of Guillain-Barré syndrome pathophysiology and the central role of the classical complement pathway in axonal injury

**DOI:** 10.3389/fneur.2025.1572949

**Published:** 2025-05-14

**Authors:** Kenneth C. Gorson

**Affiliations:** Department of Neurology, St Elizabeth’s Medical Center, Tufts University School of Medicine, Boston, MA, United States

**Keywords:** Guillain-Barré syndrome, classical complement, C1q, pathophysiology, acute motor axonal neuropathy (AMAN), acute inflammatory demyelinating polyneuropathy (AIDP), ANX005, tanruprubart

## Abstract

Guillain-Barré syndrome (GBS) is a rare, frequently postinfectious neuromuscular emergency and the leading cause of acute paralytic neuropathy worldwide. GBS incidence varies considerably across geographic regions, owing predominantly to different infectious exposures. In GBS, antecedent infection leads to production of immunoglobulin G and immunoglobulin M antibodies that cross-react with the myelin sheath and axons of peripheral nerves. These antibodies activate the classical complement pathway, which plays a key role in peripheral nerve injury regardless of autoantibody binding to myelin or axons as a target. The heterogeneous clinical presentation and progression of GBS symptoms have long been attributed to binary axonal and demyelinating neurophysiologic classifications; however, evolving evidence indicates that these pathophysiologic processes overlap. Intravenous immunoglobulin and plasma exchange, the current standard-of-care therapies in GBS, both reduce autoantibody levels and complement activation, thereby aiming to address this convergence of pathophysiology. However, these therapies only partially decrease antibody levels and complement activity and require extended courses of treatment (5 days for intravenous immunoglobulin and 7–14 days for plasma exchange), limiting their effectiveness in addressing acute neuronal damage during the active phase of disease. Given its evolutionary role in antibody binding and activating the classical complement pathway, the complement component C1q has been proposed as a therapeutic target in GBS. The clinical trial program of the C1q inhibitor ANX005, including placebo-controlled, double-blind phase 1b and phase 3 trials in GBS, provides insight into the pathophysiology of GBS and the efficacy of C1q inhibition regardless of neurophysiologic classification or geographic location.

## 1 Introduction

Guillain-Barré syndrome (GBS) is a neuromuscular emergency and the most common and severe acute paralytic neuropathy worldwide (1, 2). The global incidence of GBS ranges from 0.30 to 6.08 cases per 100,000 population depending on geography, age, exposures to infections, and other risk factors (3, 4). The incidence is especially high in low- and middle-income countries, including Bangladesh and the Philippines (5, 6).

In most cases, GBS occurs following an antecedent infection in a previously healthy patient. The disease often begins with muscle weakness that rapidly progresses over a few days and, in up to 30% of patients, can lead to respiratory failure requiring mechanical ventilation and intensive care admission (2, 7, 8). Patients may also present with sensory symptoms, such as pain and paresthesia, as well autonomic dysfunction that may result in life-threatening hemodynamic instability or arrhythmias (1, 2). Mortality rates range from 3 to 10% (1, 9–11) and are highest among those who require mechanical ventilation (12, 13). Although most deaths in GBS occur in the first 6 months after disease onset, mortality risk may be increased for several years beyond the acute phase of illness and does not appear to be influenced by current treatments (1, 14, 15).

Many patients with GBS who achieve good functional recovery following the acute phase of disease may still experience prolonged pain and fatigue (2). The prevalence of residual pain and other sensory deficits varies from 33% to 82% depending on symptom location, muscle group (for those with residual chronic muscle pain), and subtype of sensory deficit (e.g., paresthesia, dysesthesias, radicular or neuropathic pain, allodynia) (16–20). Sensory deficits are associated with disruptions in quality of life, while pain intensity correlates with disability, strength impairment, and fatigue (16, 19). As with disease incidence, mortality and other outcomes in GBS may vary regionally, based on extrinsic factors, including access to health care resources (6, 9, 21, 22).

GBS is a prototypical postinfectious autoimmune disorder (2, 23), and *Campylobacter jejuni* is the most prevalent antecedent infection worldwide (24, 25). The prevailing evidence strongly supports molecular mimicry as the pathogenic mechanism of disease (26, 27). Antibodies that are generated against *C jejuni* or other infectious agents cross-react with antigens in peripheral nerve axons and myelin (27, 28). Serum immunoglobulin (Ig) M and IgG antibodies against gangliosides or glycolipid complexes are detected in up to 92% of patients with GBS during the acute, active phase of disease, and their levels subsequently decline in the following weeks (27, 29–31). Regardless of the antecedent infection, the cross-reactive autoantibodies engage the classical complement pathway, which drives the peripheral nerve injury and neuroinflammation underlying disease pathology (23, 27, 28, 32–34).

The heterogeneous clinical presentation and progression of symptoms in GBS may lead to diagnostic delay during the active phase of the disease, further underscoring the need for a treatment that rapidly and completely inhibits key pathophysiologic components of disease once the diagnosis is confirmed.

## 2 Pathophysiology of GBS

### 2.1 Reappraisal of the distinction between axonal and demyelinating mechanisms in GBS

The variability in prognosis and long-term recovery in GBS has historically been attributed to the specific site of peripheral nerve damage, as characterized by nerve conduction studies (NCSs). In this classification, the primary targets of immune-mediated attack are either the axonal components of peripheral nerves, leading to acute motor axonal neuropathy (AMAN), or the myelin sheath (produced by Schwann cells), resulting in acute inflammatory demyelinating polyneuropathy (AIDP). AMAN is often associated with more severe disease (27, 35). The relative frequencies of each NCS subtype vary across geographic regions, potentially due to differing infectious exposures or genetic susceptibilities (27, 36).

Despite the dichotomized NCS classifications, the diagnostic criteria for GBS are identical for AMAN and AIDP, and neither classification dictates treatment strategies or prognosis (37–43). While earlier studies suggested that patients with AMAN experience a worse prognosis compared with those with AIDP (12, 27), more recent research indicates that other factors such as baseline severity of weakness, as measured by Medical Research Council (MRC) scale sum score, and serum and cerebrospinal fluid (CSF) neurofilament light chain (NfL) and neurofilament heavy chain levels during active disease are more relevant in predicting outcome in patients with GBS (12, 44–48). Acutely elevated serum NfL levels reflect the level of axonal damage in early disease and are elevated in both the axonal and demyelinating NCS subtypes, indicating varying degrees of axonal involvement, even in those patients with a primarily demyelinating presentation (45).

Axonal loss has been demonstrated in AIDP as well as AMAN and is the main determinant of long-term disability (35, 48, 49). Ultimately, electrodiagnostic studies often reveal a mixed pattern of demyelination and axonal damage or yield inconclusive results. In a retrospective study of patients with very early-stage GBS who had initial NCSs performed within 4 days of symptom onset, classification was possible in only 20% of patients. Specifically, 40% showed a mixed pattern of demyelination and axonal degeneration on NCSs, and 33% showed equivocal results (50). Similarly, in a study of patients with AIDP assessed within 10 days of symptom onset, NCSs often showed nonspecific abnormalities (51). Furthermore, the classification of electrodiagnostic findings in GBS appears to vary based on which NCS criteria are used. In an analysis of 1,137 NCSs from the first 1,500 patients enrolled in the International GBS Outcome Study, only 68% were classified identically according to the Hadden and Rajabally criteria, which are among the most commonly used NCS criteria in GBS research (52). NCS abnormalities are typically not confirmed via pathologic material (i.e., nerve biopsy), further complicating these seemingly artificial distinctions.

Given these limitations, a reappraisal of the oversimplified classification of GBS into axonal and demyelinating forms is warranted from diagnostic, pathophysiologic, and prognostic standpoints. The evolving understanding of common pathways in GBS pathophysiology further challenges the application of rigid electrophysiologic classifications when considering current and future treatment options. Regardless of NCS findings, GBS represents a neuromuscular emergency (1, 2, 39, 43), in which rapid, effective treatment is required to prevent acute and ongoing damage to axons and mitigate neuroinflammation (2, 8, 42, 53). As research advances in GBS and treatment becomes further personalized, pathophysiological differences between AMAN and AIDP which are clinically relevant may be uncovered or targeted with new treatments. However, the convergence of axonal loss in both NCS classifications also suggests that a unified, targeted treatment approach that addresses complement-mediated nerve damage driven by autoantibodies against either myelin or axons can be effective for all patients with GBS.

### 2.2 Revelation of key mechanistic characteristics in GBS based on current standard-of-care therapies

Intravenous immunoglobulin (IVIg) and plasma exchange (PE) have been the standard treatments for GBS since their introduction in the 1980s (53–55). Both are believed to function by reducing circulating autoantibody levels and decreasing complement activity (27, 35, 56–59). IVIg is used in most patients with GBS, given its relative ease of administration and accessibility (54, 58, 60). However, evidence suggests that IVIg and PE are equivalent in efficacy (35, 42, 53, 55, 60, 61), consistent with their overlapping mechanisms and the core pathophysiology of GBS. Both IVIg and PE require a prolonged treatment cycle, typically spanning 5 days for IVIg and 7 to 14 days for PE for a full course of therapy (35, 42, 53, 62–64), and neither fully eliminates autoantibodies or halts complement activity (65–69).

Despite treatment with IVIg or PE, along with optimal supportive care including mechanical ventilation, a substantial proportion of patients with GBS experience persistent symptoms (1, 2, 58). Resolution of the disease is rare in the first week of treatment (9), and up to 50% of patients treated with IVIg or PE show no improvement on the GBS Disability Scale (GBS-DS) at 4 weeks (70). Even with time, only a minority of patients with GBS achieve a GBS-DS score of 0, indicating a return to pre-disease levels of function or being healthy (15, 17, 18, 71). Moreover, even among those who regain functional abilities, including restored ambulation, many continue to face challenges with walking, experience limitations in activities of daily living, including work and hobbies, and report suboptimal health-related quality of life (2, 72–77).

Global expert consensus emphasizes the importance of early treatment to achieve maximum therapeutic benefits and avert irreversible axonal damage in this rapidly progressive disease (1, 2, 27, 42, 53, 78, 79). A recent study involving 136 IVIg-treated patients found that shorter time to treatment initiation was associated with better outcomes regardless of NCS pattern, with the ideal treatment window being within the first 2 weeks (79). Early treatment becomes even more critical given that IVIg and PE tend to act slowly and require several days to complete a full course of therapy.

Although as many as a third of patients with GBS receive additional treatment after the initial intervention, owing to persistent deterioration or lack of improvement after first-line therapy (60, 80), current evidence does not support this practice. There is no proven benefit to combining or sequentially administering IVIg and PE (42, 58, 62, 70, 81), and repeated dosing of either therapy has not demonstrated superior outcomes relative to single dosing (82–84). For instance, a second dose of IVIg did not provide additional benefit over placebo and was associated with a higher frequency of serious adverse events in patients with severe GBS who deteriorated 1 week after their first course of IVIg (84). Similarly, increasing the number of PE sessions from 4 to 6 did not yield better results in patients with severe GBS (82).

Ultimately, findings from studies evaluating alternative regimens for IVIg and PE suggest that the full therapeutic potential of these therapies in GBS has been realized, highlighting a substantial unmet medical need for therapies capable of rapidly preventing acute and ongoing nerve damage and axonal loss. Moreover, it appears that the treatment window in GBS is relatively short, necessitating an intervention that acts rapidly to prevent further deterioration that limits the ability to recover.

### 2.3 Central role of classical complement activation in GBS pathophysiology

Complement represents a distinct pathway in GBS that mediates rapid tissue damage and destruction and inflammation (23, 27, 32, 33). When the pathway is engaged on the cell surface, it activates within seconds to coat the surface with complement activation products; these products cause direct membrane damage or destruction and recruit inflammatory macrophages that destroy tissue (1, 27, 33, 34, 36, 85). Anaphylatoxins released during classical complement activation drive neuroinflammation by activating a number of inflammatory cell types, including macrophages (33, 34, 86, 87). This mechanism is consistent with the rapid course of disease in GBS that can trigger pain, paralysis, and even death within a period of days (8, 11). In a large cohort of 567 patients with GBS from multiple clinical trials, 80% of patients reached clinical nadir (i.e., maximum disability or muscle weakness) within 2 weeks following the onset of weakness, and 97% reached their nadir within 4 weeks (88).

**Figure 1:**
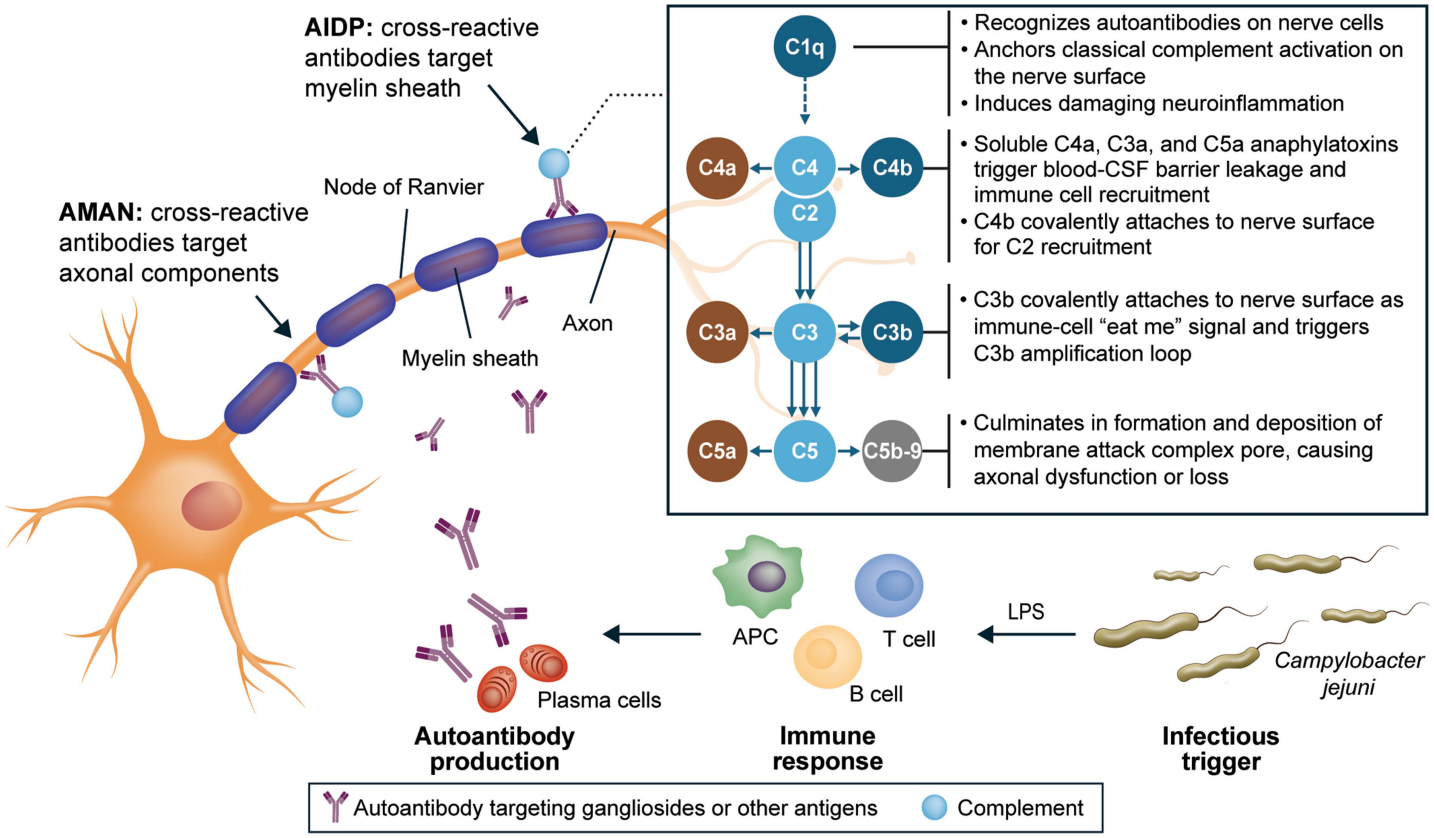
Integral role of C1q in neuroinflammation and nerve damage in GBS (23, 27, 32–34, 91–94). AIDP, acute inflammatory demyelinating polyneuropathy; AMAN, acute motor axonal neuropathy; APC, antigen-presenting cell; C, complement component; CSF, cerebrospinal fluid; GBS, Guillain-Barré syndrome; LPS, lipopolysaccharide.

The complement system functions similarly in all individuals, independent of their genetic or ethnic background, because it is an evolutionarily conserved mechanism designed to recognize and eliminate pathogens by using pattern recognition (34, 89). The high conservation of complement components C1q, C2, C3, C4, and C5-C9 and their activities across species underscores the essential and nonredundant roles of these universal processes in ensuring rapid immune responses (90). In GBS, binding of C1q to antibodies bound to peripheral nerves activates the classical complement pathway directly on the nerve surface, mediating a cascade of events that culminates in neuroinflammation and nerve damage and destruction (Figure 1) (23, 27, 32–34, 91–94). Evidence also suggests that classical complement activation underlies nerve injury in GBS regardless of axonal or demyelinating pathophysiology (27, 28, 33, 36, 85). Given the early role of complement activation in the pathophysiology of GBS, complement inhibition is a potentially effective target for halting significant axonal damage in the acute phase of the disease and for optimizing long-term function.

## 3 Insights from a C1q inhibitor clinical development program in GBS

ANX005, a humanized monoclonal antibody, is designed to inhibit C1q selectively, rapidly, and fully, thus preventing initiation of the classical complement cascade (32). Blocking C1q at the start of the classical complement pathway inhibits tissue inflammation and damage driven by downstream complement components, including C3a and C5a (soluble anaphylatoxins that are highly proinflammatory), C4b and C3b (cell surface phagocytosis signals), and C5b-9 (which causes direct membrane damage; Figure 1) (32–34, 86, 87). Furthermore, due to the nature of the complement system with C1q as its initiating molecule, the biological mechanisms of ANX005 are expected to be consistent regardless of a patient’s race, ethnicity, or geographic location. The ANX005 development program in GBS consists of 2 placebo-controlled, double-blind clinical trials, a phase 1b trial and a phase 3 trial (NCT04701164), designed to evaluate the safety and efficacy of a single dose of ANX005 as a treatment for GBS. The phase 1b trial was conducted in Bangladesh, and the phase 3 trial was conducted in Bangladesh and the Philippines.

Among patients in the phase 1b trial with antibodies against the GM1 ganglioside in CSF (including patients with either AMAN or AIDP), complement deposition on GM1-coated plates was rapidly reduced in those treated with a single dose of ANX005 vs. placebo, consistent with complete inhibition of C1q (95). An analysis of data from this study also provided valuable insight into the coexistence of axonal damage and demyelination in GBS pathogenesis (96). Patients exhibited elevated levels of biomarkers of both axonal damage (NfL) (48) and demyelination (CSF sphingomyelin) (97) regardless of the NCS pattern. Peak NfL levels inversely correlated with MRC scores, and patients with lower NfL levels at baseline were more likely to show improvement on GBS-DS score at week 8, a consistent observation regardless of axonal or demyelinating physiology. Furthermore, and also independent of NCS findings, NfL level was the most important predictor of GBS-DS score at week 8 based on random forest modeling of multiple prognostic biomarkers and clinical characteristics, followed by CSF sphingomyelin level. The results of this study support a common pathophysiologic pathway across both axonal and demyelinating NCS patterns and suggest the effectiveness of complement inhibition in all patients with GBS (96).

In the subsequent phase 3 trial, patients who received a single 30-mg/kg ANX005 infusion showed complete inhibition of complement on the first day of administration and experienced rapid and sustained improvements in function and health status compared with those receiving placebo. Significant response was seen as early as week 1 in MRC sum score change from baseline (*p* < 0.0001) compared to placebo, and ANX005 30-mg/kg–treated participants were able to discontinue mechanical ventilation 28 days earlier and walk independently 31 days sooner than participants receiving placebo. This response was maintained, with a greater percentage in the ANX005 30-mg/kg group returning to a GBS-DS score of 0 at 26 weeks (22% vs. 9% in the placebo group; odds ratio, 4.1; 95% CI, 1.42–12.04; *p* = 0.0092). Significant response was maintained in patients with AMAN and AIDP and in subgroups with baseline MRC sum score and NfL levels typical of approximately 80% of patients with GBS in the United States and Europe. Hence, the results of this study are expected to be generalizable to patients in those regions. ANX005 was also well tolerated, with infusion-related reactions being the most common adverse events (98).

## 4 Discussion

GBS stands as the most prevalent and severe acute paralytic neuropathy globally, presenting significant challenges in diagnosis, treatment, and long-term management. The heterogeneous clinical manifestations and variable prognosis of this disease are rooted in its complex pathophysiology, primarily driven by autoantibody-mediated complement activation that results in both axonal damage and demyelination. Standard therapies, namely IVIg and PE, have been instrumental in the management of GBS; however, they are constrained by their delayed effect, prolonged treatment courses, and failure to consistently prevent residual symptoms such as pain, fatigue, and functional disabilities. Moreover, the critical window for effective intervention in GBS is notably short, underscoring the urgent need for treatments that act rapidly to halt disease progression and mitigate irreversible nerve damage.

Recent advancements in the understanding of GBS pathophysiology have highlighted the central role of classical complement activation in driving neuroinflammation and nerve damage and destruction, regardless of NCS pattern. This recognition has paved the way for targeted therapeutic approaches, such as the development of ANX005, a humanized monoclonal antibody designed to inhibit C1q selectively. Clinical trials have demonstrated that a single dose of ANX005 not only effectively suppressed complement activation but also yielded significant and sustained improvements in functional outcomes in patients with GBS, independent of NCS subtype. These findings validate the hypothesis that complement inhibition can serve as a unifying therapeutic strategy, addressing the underlying mechanisms common to all patients with GBS.

The success of ANX005 in clinical trials emphasizes the necessity of moving beyond traditional binary classifications of GBS and adopting a more nuanced understanding of its pathogenesis. By targeting universal pathogenic pathways, ANX005 offers a promising solution to the unmet medical need for rapid and comprehensive treatments that can prevent both acute and ongoing nerve damage. However, new treatments may also come with other limitations, such as limited initial accessibility and clinical experience.

While IVIg and PE have established a foundation for GBS management, their limitations highlight the pressing demand for novel therapies that can deliver faster and more complete therapeutic benefits. The development and validation of complement inhibitors like ANX005 represent a significant breakthrough, offering hope for improved outcomes and enhanced quality of life for patients afflicted by this debilitating neuromuscular emergency. As our understanding of GBS continues to evolve, embracing targeted treatments that address its core pathophysiologic mechanisms will be pivotal in transforming the landscape of GBS care and patient recovery.

## Data Availability

The original contributions presented in the commentary are included in the article/supplementary material, further inquiries can be directed to the corresponding author.
